# Nanoscale coupling of junctophilin-2 and ryanodine receptors regulates vascular smooth muscle cell contractility

**DOI:** 10.1073/pnas.1911304116

**Published:** 2019-10-07

**Authors:** Harry A. T. Pritchard, Caoimhin S. Griffin, Evan Yamasaki, Pratish Thakore, Conor Lane, Adam S. Greenstein, Scott Earley

**Affiliations:** ^a^Department of Pharmacology, Center for Molecular and Cellular Signaling in the Cardiovascular System, University of Nevada, Reno School of Medicine, Reno, NV 89557;; ^b^Division of Cardiovascular Sciences, Faculty of Biology, Medicine and Health, University of Manchester, Manchester M13 9NT, United Kingdom

**Keywords:** ion channels, cerebral arteries, electrophysiology, super-resolution microscopy, Ca^2+^ signaling

## Abstract

Junctophilin proteins maintain close contacts between the endoplasmic/sarcoplasmic reticulum (ER/SR) and the plasma membrane in many types of cells, as typified by junctophilin-2 (JPH2), which is necessary for the formation of the cardiac dyad. Here, we report that JPH2 is the most abundant junctophilin isotype in native smooth muscle cells (SMCs) isolated from cerebral arteries and that acute knockdown diminishes the area of sites of interaction between the SR and plasma membrane. Superresolution microscopy revealed nanometer-scale colocalization of JPH2 clusters with type 2 ryanodine receptor (RyR2) clusters near the cell surface. Knockdown of JPH2 had no effect on the frequency, amplitude, or kinetics of spontaneous Ca^2+^ sparks generated by transient release of Ca^2+^ from the SR through RyR2s, but it did nearly abolish Ca^2+^ spark-activated, large-conductance, Ca^2+^-activated K^+^ (BK) channel currents. We also found that JPH2 knockdown was associated with hypercontractility of intact cerebral arteries. We conclude that JPH2 maintains functional coupling between RyR2s and BK channels and is critically important for cerebral arterial function.

## Significance

The junctophilins are a family of structural proteins that organize intracellular membrane junctions, such as coupling between the endoplasmic/sarcoplasmic reticulum and plasma membrane. The junctophilins are critically important for cardiac and skeletal muscle function, but little is known about how these proteins influence vascular smooth muscle cells (SMCs). We found that one isotype, junctophilin-2 (JPH2), is abundant in SMCs and is essential for maintaining a subcellular Ca^2+^ signaling pathway that is critically important for regulation of membrane potential and contractility. Our data also show that impaired expression of JPH2 results in hypercontractility of small cerebral arteries. These findings suggest that loss-of-function mutations or decreased expression of JPH2 could contribute to vascular pathologies such as systemic hypertension and vascular cognitive impairment.

In cardiac and skeletal muscle cells, close coupling of the plasma membrane and endoplasmic/sarcoplasmic reticulum (ER/SR) is maintained in part by junctophilin proteins (1–3). All junctophilin proteins contain a transmembrane domain that anchors the C terminus within the ER/SR membrane and an N-terminal MORN (membrane occupation and recognition nexus) motif that noncovalently binds to the plasma membrane (2, 3). This unique structure allows junctophilins to tether the ER/SR with the plasma membrane, maintaining close proximity. Four unique junctophilin proteins (JPH1–4) have been described. JPH1 and JPH2 are present and functionally important in skeletal muscle (3), whereas JPH2 is the only isotype expressed in cardiac muscle (3). JPH3 and JPH4 are not present in muscle, but are expressed in central neurons (4) and other tissues (5, 6). Junctophilin proteins are necessary for the formation of signaling complexes within transverse (T) tubules of cardiac and skeletal muscle cells that are essential for efficient excitation–contraction (EC) coupling (3, 7, 8). Close contacts between the SR and plasma membrane form stable peripheral coupling sites in contractile vascular smooth muscle cells (SMCs) (9, 10), but little is currently known about the role of junctophilin proteins in the formation of these structures.

Subcellular domains support localized Ca^2+^ signaling pathways that are critically important for the regulation of SMC membrane potential and contractility (10). The specific functions of discrete, transient, and spatially localized changes in intracellular Ca^2+^ concentration depend on the structural arrangement of the relevant signaling elements within these domains. For example, well-characterized Ca^2+^-release events known as “Ca^2+^ sparks” are rapid, high-amplitude Ca^2+^ signals generated by the release of Ca^2+^ ions through clusters of type 2 ryanodine receptors (RyR2s) in the SR membrane (11, 12). RyR2 clusters are functionally coupled with multiple large-conductance Ca^2+^-sensitive K^+^ (BK) channels such that a single Ca^2+^ spark generates a large transient outward K^+^ current that hyperpolarizes the plasma membrane, deactivating voltage-dependent Ca^2+^ influx to cause arterial relaxation (13–15). Loss of peripheral coupling upon depolymerization of microtubules uncouples RyR2 and BK channel clusters, diminishing transient BK channel activity and vascular hypercontractility, demonstrating the functional importance of close interactions between RyR2s and BK channels (10).

In the present study, we investigated the significance of junctophilin proteins in the formation of peripheral coupling signaling domains in native SMCs and the consequences of loss of junctophilin expression on the Ca^2+^ spark–BK channel signaling pathway and vascular contractility. We found that *Jph2* is the most abundant junctophilin isotype in native SMCs obtained from cerebral resistance arteries, and that this structural protein is necessary for site-specific juxtaposition of the SR and plasma membrane at the periphery of these cells. Using superresolution microscopy, we show that JPH2 and RyR2s colocalize near the surface of native cerebral artery SMCs. We further show that, although acute knockdown of JPH2 expression has no direct effect on the frequency, amplitude, or kinetics of spontaneous Ca^2+^ sparks, the protein is essential for functional coupling of RyR2 with BK channels in cerebral artery SMCs. JPH2 knockdown resulted in hypercontractility of intact cerebral arteries, demonstrating an important role for this protein in cerebral vascular control.

## Results

### JPH2 Is Required for Peripheral Coupling of the SR and Plasma Membrane in SMCs.

**Fig. 1.**
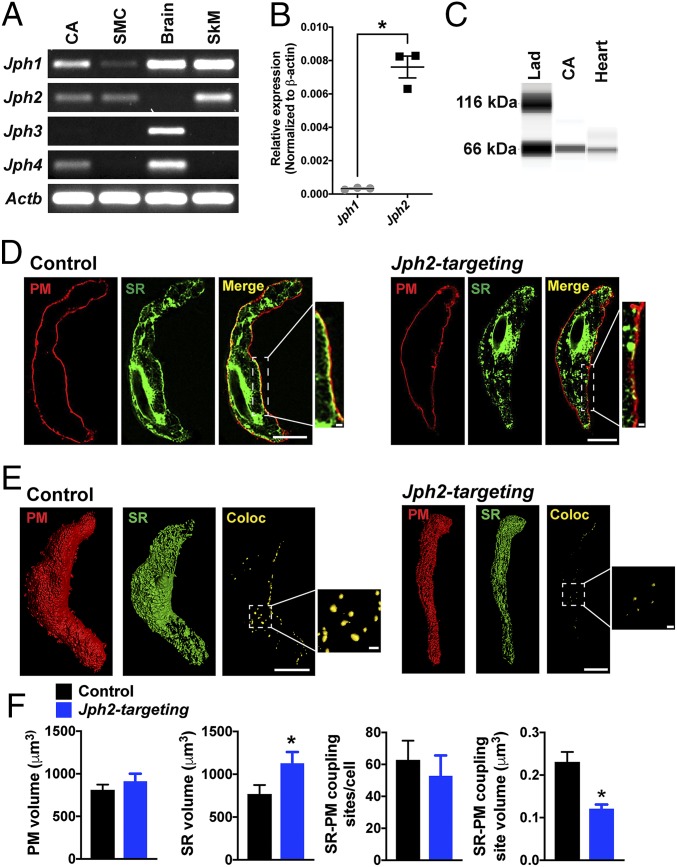
JPH2 maintains the proximity of the plasma membrane and SR in cerebral artery SMCs. (*A*) Representative end-point RT-PCR analysis for expression of *Jph1, Jph2*, *Jph3*, and *Jph4* in RNA samples isolated from whole cerebral arteries (CA), and in SMCs isolated from cerebral arteries, whole brain, and skeletal muscle (SkM; *n* = 3 independent experiments). β-Actin (*Actb*) was used as a positive control. (*B*) Relative expression levels of *Jph1* and *Jph2* mRNAs from isolated SMCs, normalized to *Actb* expression (*n* = 3; *P < 0.05). (*C*) Representative Wes protein analysis of whole cerebral artery (CA) and heart lysates probed with an anti-JPH2 antibody. (*D*) Deconvolved confocal slice of isolated SMCs from cerebral arteries treated with control or *Jph2*-targeting morpholinos and stained with dyes that specifically target the plasma membrane (PM; red) or SR (green). Merged images show the effects of morpholino treatment on close interactions of the PM and SR at the periphery of the cell in respective insets. (Scale bars: full images, 10 µm; *Insets*, 1 µm.) (*E*) Representative surface analysis of *z*-stack reconstructions of SMCs isolated from cerebral arteries treated with control or *Jph2*-targeting morpholinos and labeled with dyes staining the PM (red) or SR (green). Colocalization surface representations (Coloc; yellow) were generated from voxels that were positive for both channels. (Scale bar, 10 µm.) (*Insets*) Magnified views of colocalizing surfaces. (Scale bar, 1 µm.) (*F*) Summary data showing mean PM and SR volumes, the number of PM–SR colocalizing sites per cell, and the mean volume of individual PM–SR colocalizing sites in SMCs isolated from cerebral arteries treated with control or *Jph2*-targeting morpholinos (*n* = 634 to 817 individual colocalization sites in *n* = 12 to 13 cells per group from 3 animals; *P < 0.05).

End-point reverse-transcription PCR (RT-PCR) was used to determine if transcripts encoding junctophilin proteins are present in cerebral and mesenteric resistance arteries isolated from adult mice. Transcripts corresponding to *Jph1*, *Jph2*, and *Jph4* were detected in RNA samples prepared from whole cerebral arteries (Fig. 1*A*). *Jph1*, *Jph2*, and *Jph3* were detected in RNA from whole mesenteric arteries (*SI Appendix*, Fig. S1). *Jph1* and *Jph2* transcripts were also detected in RNA obtained from enriched pools of SMCs isolated by enzymatic dispersal of intact cerebral arteries followed by fluorescence-activated cell-sorting (Fig. 1*A*). Expression of *Jph3* was not detected in RNA prepared from whole cerebral arteries or isolated SMCs, but was present in RNA samples isolated from whole brain (Fig. 1*A*). These data suggest that *Jph1* and *Jph2* are present in SMCs, and that other *Jph* isotypes may be expressed in other cell types present in whole arteries, such as fibroblasts, pericytes, and/or endothelial cells. A comparison of expression levels of *Jph1* and *Jph2* using quantitative RT-PCR (RT-qPCR) showed that *Jph2* expression was ∼24-fold greater than *Jph1* expression in SMCs isolated from native cerebral arteries (Fig. 1*B*), indicating that *Jph2* is the most abundant junctophilin in contractile SMCs. Expression of JPH2 protein in cerebral arteries was investigated using the Wes capillary electrophoresis immunoassay system (Fig. 1*C*). Protein lysates from whole cerebral arteries and hearts probed with a primary antibody against JPH2 produced bands at the predicted molecular weight of the protein (∼66 kDa).

We focused the remainder of our study on *Jph2* because it is the most abundant junctophilin isotype in vascular SMCs. Global knockout of *Jph2* is lethal during embryotic development, probably due to cardiac dysfunction (3). Therefore, we used an acute knockdown approach to determine the function of JPH2 in native SMCs. Using an established protocol (16, 17), we treated isolated cerebral arteries with silencing oligonucleotides (morpholinos) targeting *Jph2* or control (nonsilencing) morpholinos and cultured them in serum-free media for 48 h. The effects of this treatment were assessed by comparing JPH2 protein levels in lysates obtained from arteries treated with *Jph2*-targeting morpholinos with those obtained from arteries treated with control morpholinos using Wes protein analysis. The area under the electropherogram curve at 66 kDa (the predicted molecular weight of JPH2) for each sample probed with a primary antibody against JPH2 was normalized to that for the same sample probed with primary antibody against β-actin (*SI Appendix*, Fig. S2). Using this approach, we found that treatment with *Jph2*-targeting morpholinos decreased JPH2 protein expression by ∼50% compared with tissues treated with control morpholinos (*SI Appendix*, Fig. S2).

To visualize the spatial arrangement of the SR and plasma membrane, we loaded SMCs isolated from arteries treated with *Jph2*-targeting or control morpholinos with CellMask Deep Red and ER-Tracker Green, which selectively label the plasma membrane and SR, respectively (Fig. 1*D*), as described in our prior publication (10). Confocal images of SMCs from arteries treated with control morpholinos loaded in this manner clearly show the plasma membrane outlining the cell and the structure of the SR within (Fig. 1*D*). Two segments of the SR are apparent: (i) the central SR in the perinuclear region and (ii) the peripheral SR near the plasma membrane. Merged images show extensive areas of close association of the peripheral SR and plasma membrane in SMCs treated with control morpholinos, but coupling of the 2 membranes was less obvious in cells treated with *Jph2*-targeting morpholinos (Fig. 1*D*). Deconvolved *z*-stack confocal images were reconstructed, and 3-dimensional (3D) representations of the plasma membrane (Fig. 1*D*, red) and SR (Fig. 1*D*, green) were generated; a third surface plot showing colocalized voxels in yellow was also created (Fig. 1*E* and Movies S1 and S2). An analysis of these images showed that total plasma membrane volume did not differ between SMCs isolated from arteries treated with control and those treated with *Jph2*-targeting morpholinos, but did reveal a significant increase in the SR volume in SMCs isolated from arteries treated with *Jph2*-targeting morpholinos compared with controls (Fig. 1*F*). We also found that, although the number of sites of interaction between the plasma membrane and SR per cell did not differ between arteries treated with control and *Jph2*-targeting morpholinos (Fig. 1*E*, yellow puncta), *Jph2* knockdown dramatically reduced the surface area of individual coupling sites (Fig. 1 *E* and *F*). These data demonstrate that acute knockdown of JPH2 significantly reduces the area of discrete sites of colocalization between the plasma membrane and SR in contractile SMCs, suggesting that JPH2 is critically important for maintaining these interactions.

### Nanoscale Colocalization of JPH2 and RyR2 in SMCs.

**Fig. 2.**
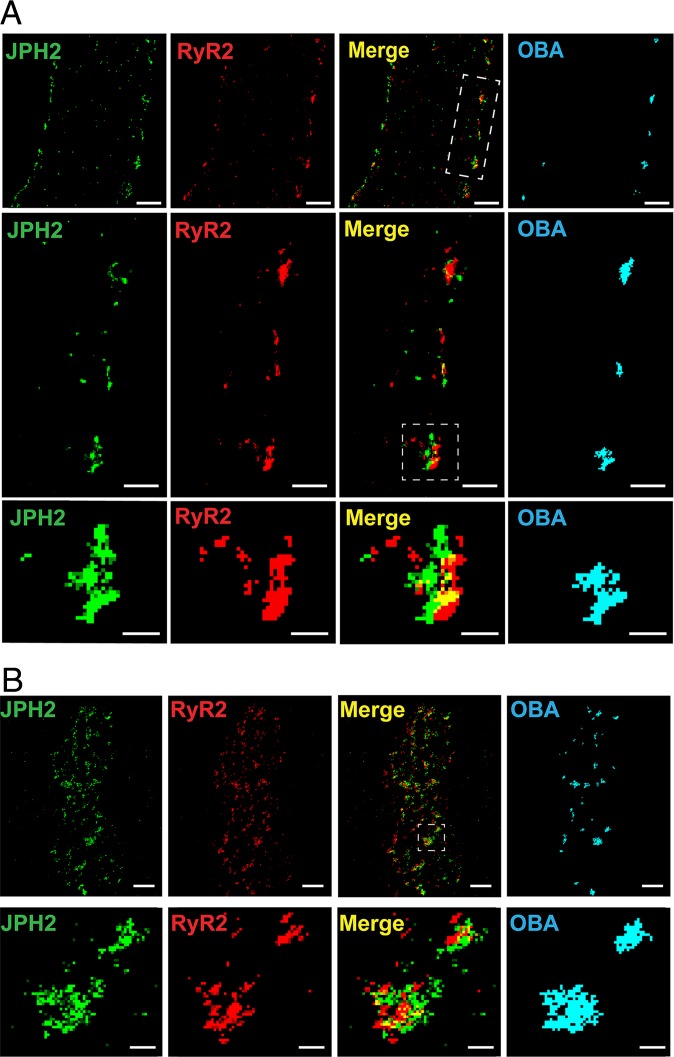
Nanometer-scale colocalization of JPH2 and RyR2 at the plasma membrane of cerebral artery SMCs. (*A*) Superresolution localization maps for a native cerebral artery SMC immunolabeled for JPH2 (green) and RyR2 (red) imaged using GSDIM in epifluorescence illumination mode. Merged images and colocalized protein clusters identified by object-based analysis (OBA) are also shown. (Scale bar, 1 µm.) The middle row shows an expanded view of the white rectangle from the top merged panel. (Scale bar, 0.5 µm.) The bottom row is a further expanded view of an interacting cluster (white box). (Scale bar, 0.2 µm.) Maps are representative of *n* = 9 cells from 3 animals. (*B*) Superresolution localization maps for a native cerebral artery SMCs immunolabeled for JPH2 (green) and RyR2 (red) imaged using GSDIM in TIRF illumination mode. (Scale bar, 1 µm.) The bottom row shows an expanded view of clusters interacting in the white square from the merged panel. (Scale bar, 0.2 µm.) Maps are representative of *n* = 7 cells from 3 animals.

Prior studies have reported that JPH2 and RyR2 protein clusters colocalize in T-tubules and peripheral coupling sites in cardiomyocytes (18–20). We therefore investigated colocalization of JPH2 and RyR2 in native cerebral artery SMCs using the superresolution modality GSDIM (ground-state depletion followed by individual molecule return) (21). SMCs were coimmunolabeled for JPH2 and RyR2 and imaged using GSDIM in the epifluorescence illumination mode. The resulting superresolution localization maps showed that both proteins are present as clusters of discrete sizes that appeared to be more abundant at the cellular periphery (Fig. 2*A*). Cluster size was exponentially distributed for both proteins (*SI Appendix*, Fig. S3 *A* and *B*). This observation is consistent with a model suggesting that membrane-associated protein clusters (including RyR2 and JPH2) are formed by stochastic self-assembly and their size is maintained by a relatively rapid rate of turnover (22). Using object-based analysis (23) to identify protein clusters that are juxtaposed within the limit of resolution of our GSDIM system (<40 nm; *SI Appendix*, Fig. S4), we found extensive areas of JPH2 and RyR2 cluster colocalization that were almost exclusively localized to the cellular periphery (Fig. 2*A*). The fraction of colocalizing clusters was significantly greater than that for a simulated random distribution of cluster localization (*SI Appendix*, Fig. S3*C*). SMCs coimmunolabeled for JPH2 and RyR2 were also imaged using GSDIM in total internal reflection fluorescence (TIRF) mode, a technique that exclusively illuminates structures within an evanescent field encompassing a cytosolic depth of only ∼150 nm from the plasma membrane. Object-based analysis of images obtained using TIRF-mode GSDIM also showed extensive areas of colocalization of JPH2 and RyR2 protein clusters near the cell surface (Fig. 2*B*). Thus, the fraction of colocalizing clusters was significantly greater than that for a simulated random distribution (*SI Appendix*, Fig. S3*D*).

### Knockdown of JPH2 Expression Has No Direct Effect on Ca^2+^ Sparks, Total SR Ca^2+^ Store Load, or SR Ca^2+^ Uptake.

**Fig. 3.**
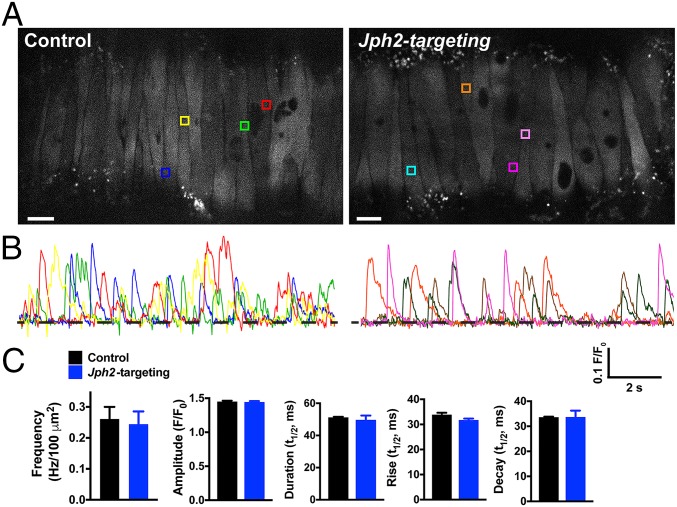
*Jph2* knockdown has no effect on Ca^2+^ spark frequency, amplitude, or kinetics. (*A*) Representative confocal Ca^2+^ images of pressurized (60 mmHg), Fluo-4-AM–loaded cerebral arteries treated with control or *Jph2*-targeting morpholinos. Colored boxes show selected ROIs where Ca^2+^ sparks occurred. (Scale bar, 10 µm.) (*B*) Representative changes in fractional fluorescence (*F*/*F*_0_) as a function of time for ROIs in *A*. The trace color corresponds to the color of the respective ROI box. (*C*) Summary data showing the Ca^2+^ spark frequency (in Hertz) normalized to surface area (in Hertz per 100 square micrometers) in cerebral arteries (*n* = 5 to 6 cerebral arteries/group from 4 animals), as well as the amplitude (*F*/*F*_0_), half-duration [half-time (*t*_1/2_), in seconds], rise time (*t*_1/2_, in seconds), and decay time (*t*_1/2_, in seconds) of individual Ca^2+^ spark events recorded from each group (*n* = 616 events for control, *n* = 601 events for *Jph2*-targeted). There were no significant differences.

To investigate the impact of JPH2 knockdown on spontaneous Ca^2+^ spark activity, we used high-speed (∼50 fps) spinning-disk confocal microscopy to image Ca^2+^ signals in pressurized (60 mmHg), intact cerebral arteries treated with *Jph2*-targeting or control morpholinos and loaded with the Ca^2+^ indicator dye Fluo-4-AM (Fig. 3 and Movies S3 and S4). Under these conditions, highly localized, transient Ca^2+^ signals with amplitudes and kinetics similar to those reported for Ca^2+^ sparks in a prior study were frequently observed (24). Autodetection and analysis of these events demonstrated that there was no significant difference in Ca^2+^ spark frequency, mean amplitude, signal duration, or rise and decay time between the groups (Fig. 3). These data indicate that knockdown of JPH2 does not directly influence the frequency of spontaneous Ca^2+^ sparks in SMCs or alter the basic properties of individual Ca^2+^ signals.

To determine the effects of *Jph2* knockdown on SR Ca^2+^ store load, mobilization, and refilling, we monitored global changes in SMC [Ca^2+^] in response to activation of RyRs by repeated bolus administration of caffeine (10 mM). We found that the amplitude and kinetics of global Ca^2+^ signals stimulated by caffeine pulses did not significantly differ between 4 consecutive caffeine challenges and did not differ between arteries treated with control or *Jph2*-targeting morpholinos (*SI Appendix*, Fig. S6*A*). To investigate the effects of IP_3_R-mediated Ca^2+^ release, we performed similar experiments using repeated bolus administration of the purinergic receptor agonist UTP (30 μM), which acts through a G_q_-coupled signaling pathway to stimulate IP_3_-mediated Ca^2+^ release. We found that the amplitude and kinetics of UTP-induced Ca^2+^ signals did not differ between repeated trials and that the responses did not differ between arteries treated with control or *Jph2*-targeting morpholinos (*SI Appendix*, Fig. S6*B*). These data demonstrate that down-regulation of *Jph2* does not affect the mobilization of SR Ca^2+^ through RyRs or IP_3_R, does not alter SR Ca^2+^ store load, and does not impair SR Ca^2+^ uptake.

### JPH2 Is Required for Functional Coupling of RyR2 with BK Channels.

**Fig. 4.**
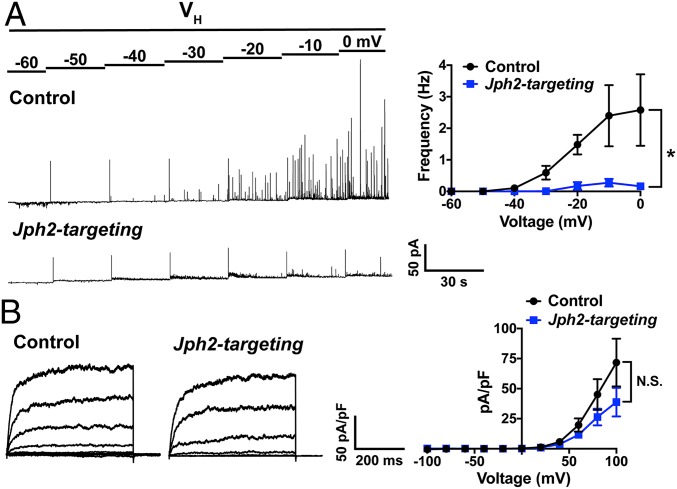
JPH2 is required for functional coupling of RyR2 and BK. (*A*) Representative recordings of STOCs in SMCs isolated from cerebral arteries treated with control or *Jph2*-targeting morpholinos recorded over a range of membrane potentials (−60 to 0 mV) using perforated patch-clamp electrophysiology. Summary data show the frequency (in Hertz) of STOCs as a function of membrane potential (*n* = 5 to 6 cells per group from 3 animals; *P < 0.05). (*B*) Representative BK (paxilline-sensitive) currents recorded from SMCs isolated from cerebral arteries treated with control or *Jph2*-targeting morpholinos using conventional whole-cell patch-clamp electrophysiology. BK currents were recorded over a series of command voltage steps (−100 to +100 mV). Summary of whole-cell current data (*n* = 8 cells/group from 4 animals). There were no significant differences.

Our data indicate that down-regulation of JPH2 expression in SMCs reduces the area of interaction between the SR and plasma membrane at individual coupling sites, but does not alter the frequency of spontaneous Ca^2+^ sparks or the amplitude or kinetics of individual Ca^2+^ spark events. We further investigated the importance of JPH2 in maintaining Ca^2+^ spark-activated BK channel activity using patch-clamp electrophysiology. SMCs isolated from arteries treated with control or *Jph2*-targeting morpholinos were patch-clamped using the amphotericin B perforated patch-clamp configuration, and Ca^2+^ spark-activated BK channel activity was recorded as spontaneous transient outward currents (STOCs) over a range of membrane potentials (−60 to 0 mV). STOCs recorded from SMCs isolated from cerebral arteries treated with control morpholinos exhibited a voltage-dependent increase in frequency. In contrast, STOCs were essentially absent from SMCs isolated from arteries treated with *Jph2*-targeting morpholinos across all membrane potentials (Fig. 4*A*). These data demonstrate that JPH2 is necessary for the generation of STOCs in cerebral artery SMCs.

In control experiments, paxilline-sensitive BK channel currents were recorded from SMCs patch-clamped in the conventional whole-cell configuration over a range of membrane potentials. There was no significant difference in the mean amplitude of whole-cell BK currents between SMCs isolated from arteries treated with control or *Jph*2-targeting morpholinos at any command potential (Fig. 4*B*). These data indicate that the number of BK channels available for activation is not altered by JPH2 knockdown. Additional controls showed that mRNA expression levels of *Kcnma1* (BK channel α subunit), *Kcnmb1* (BK channel β subunit), and *Ryr2* did not differ between arteries treated with control and *Jph2*-targeting morpholinos (*SI Appendix*, Fig. S7). Further, GSDIM studies showed that *Jph2* knockdown had no effect on RyR2 or BKα cluster size distribution or cluster density (*SI Appendix*, Fig. S8). We conclude that JPH2 expression is necessary for efficient functional coupling of RyR2s and BK channels in cerebral artery SMCs.

### Knockdown of JPH2 Expression Causes Vascular Hypercontractility.

**Fig. 5.**
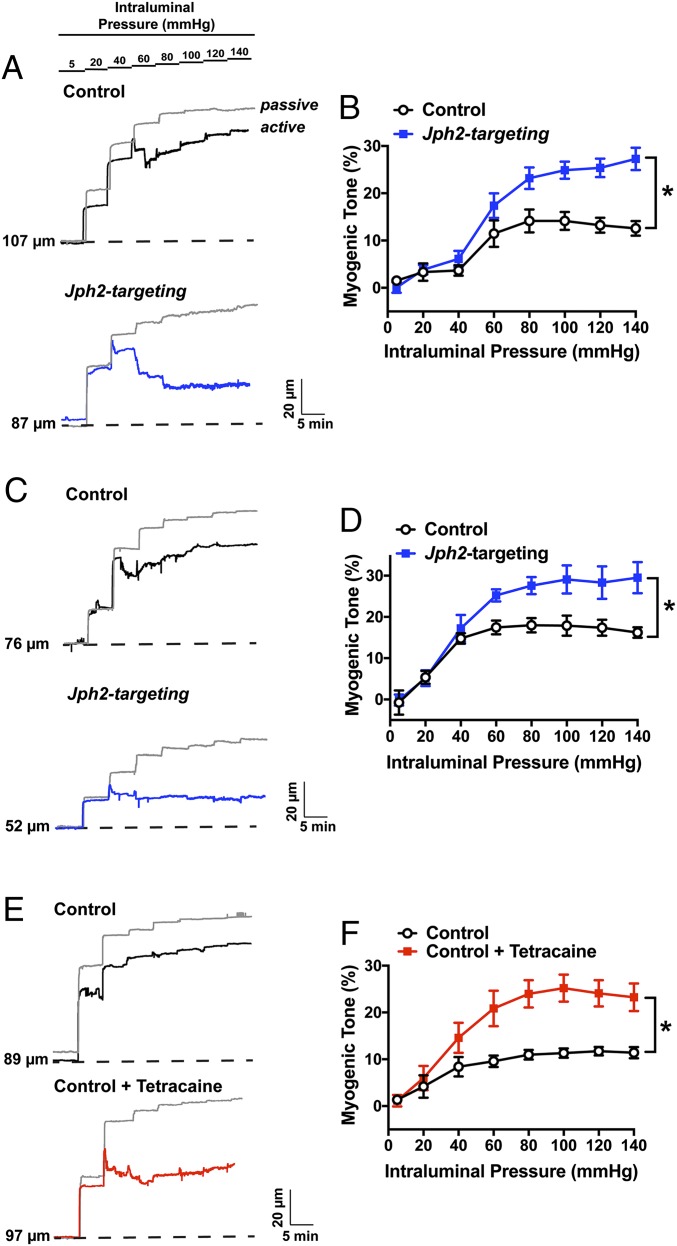
*Jph2* knockdown causes vascular hypercontractility. (*A*) Representative traces showing changes in luminal diameter over a range of intraluminal pressures (5 to 140 mmHg) for cerebral arteries treated with control (black) or *Jph2*-targeting (blue) morpholinos. The passive response to changes in intraluminal pressure for both arteries is indicated by the gray trace. (*B*) Summary data for myogenic tone as a function of intraluminal pressure for both groups (*n* = 6 arteries per group from 4 to 5 animals; *P < 0.05). (*C*) Representative traces showing changes in luminal diameter over a range of intraluminal pressures (5 to 140 mmHg) for mesenteric arteries treated with control (black) or *Jph2*-targeting (blue) morpholinos. The passive response to changes in intraluminal pressure for both groups is indicated by the gray trace. (*D*) Summary data for myogenic tone as a function of intraluminal pressure for both groups (*n* = 6 arteries per group from 4 to 5 animals; *P < 0.05). (*E*) Representative traces showing changes in luminal diameter over a range of intraluminal pressures (5 to 140 mmHg) for cerebral arteries treated with control morpholinos (black) or cerebral arteries treated with control morpholinos in the presence of tetracaine (10 µM; red). The passive response to changes in intraluminal pressure for both arteries is indicated by the gray trace. (*F*) Summary data for myogenic tone as a function of intraluminal pressure for both groups (*n* = 6 arteries per group from 4 to 5 animals; *P < 0.05).

BK channel currents activated by Ca^2+^ sparks provide critical negative feedback regulation that limits the magnitude and duration of cerebral artery constriction (15, 25). We predicted that, because knockdown of JPH2 expression nearly abolished STOCs in contractile SMCs, this maneuver would also increase vascular contractility. This possibility was investigated in a series of ex vivo pressure myography experiments using isolated cerebral arteries treated with control or *Jph2*-targeting morpholinos. Vasoconstriction in response to increases in vascular pressure (myogenic tone) was evaluated by raising intraluminal pressure from an initial value of 5 mmHg to 20 mmHg. We found that cerebral arteries treated with *Jph2*-targeting morpholinos generated significantly more myogenic tone at physiological levels of intraluminal pressure (60 mmHg) compared with arteries treated with control morpholinos (Fig. 5*B*). In contrast, constriction in response to direct depolarization of the plasma membrane of SMCs by increasing extracellular [K^+^] to 60 mM did not differ between arteries treated with control and *Jph2*-targeting morpholinos (32 ± 4% vs. 32 ± 2%; *n* = 6 per group), suggesting that increased contractility following JPH2 knockdown was not caused by gross alterations in voltage-dependent Ca^2+^-influx pathways or the contractile apparatus. Mesenteric arteries treated with *Jph2*-targeting morpholinos also developed significantly greater levels of myogenic tone compared with corresponding controls (Fig. 5 *C* and *D*), but constriction in response to elevated extracellular K^+^ did not differ between groups (65 ± 5% vs. 62 ± 4%; *n* = 6 per group). The increase in myogenic tone of cerebral vessels treated with *Jph2*-targeting morpholinos was comparable to that caused by addition of the RyR blocker tetracaine to the bathing solution (Fig. 5 *E* and *F*), supporting the concept that knockdown of JPH2 expression enhances vasoconstriction by uncoupling Ca^2+^ sparks from BK channels.

## Discussion

Communication between discrete sites of Ca^2+^ release on the SR and Ca^2+^-activated ion channels on the plasma membrane is vital for the regulation of SMC membrane potential and contractility. Here, we investigated the structural role of junctophilins in the formation of these signaling domains and how these proteins contribute to Ca^2+^-signaling pathways, ion channel activity, and contractile regulation of cerebral resistance arteries. We found that JPH2 is the most abundant junctophilin isotype in contractile SMCs isolated from cerebral arteries and that knockdown of JPH2 expression significantly reduced the area of sites of interaction between the plasma membrane and SR. We also showed that large clusters of nanometer-scale colocalized JPH2 and RyR2s are present close to the plasma membrane of SMCs. Knockdown of JPH2 expression had no effect on SR Ca^2+^ store load or Ca^2+^ uptake, the frequency of spontaneous Ca^2+^ sparks, or the amplitude and kinetics of individual events, but nearly eliminated transient BK channel currents activated by Ca^2+^ sparks, demonstrating that JPH2 is necessary for functional coupling of BK channels on the plasma membrane and RyR2s on the SR. Our data also showed that knockdown of JPH2 expression resulted in enhanced constriction of intact cerebral and mesenteric arteries in response to increases in intraluminal pressure. We conclude that JPH2 is necessary for the structural integrity of critically important peripheral Ca^2+^ signaling domains that regulate membrane potential and contractility in arterial SMCs.

JPH2 is essential for the organization of Ca^2+^ signaling complexes in cardiomyocyte T-tubules, which are critically important for EC coupling. That this function is essential was demonstrated by studies showing that genetic knockout of *Jph2* is embryonic-lethal due to heart failure resulting from impaired EC coupling (3) and that acute knockdown of *Jph2* expression in cardiomyocytes in vivo causes heart failure and death (7). SMCs lack the intracellular geometry imparted by the T-tubule network characteristic of cardiac muscle cells; instead, Ca^2+^ signaling activity in SMCs takes place at distributed sites of close contact between the SR and plasma membrane. The subcellular areas of SR–plasma membrane colocalization identified here using membrane-selective dyes likely represent these peripheral coupling signaling domains. Our prior study demonstrated that microtubule networks are essential for the maintenance of these sites in SMCs (10), but little else is known about the molecular architecture of these signaling domains. Here, we found that acute down-regulation of JPH2 diminished the area of individual sites of interaction between the plasma membrane and SR in contractile SMCs, but did not affect the total number of such sites. Our data indicate an important role for JPH2 in the maintenance and organization of these sites; however, these domains persist following JPH2 knockdown, albeit with a much-reduced surface area. It is possible that the persistence of these regions of membrane interaction reflects incomplete knockdown of JPH2 expression. Alternatively, other as yet unidentified molecules are involved in the initiation of peripheral coupling sites in SMCs.

Prior studies have used superresolution microscopy to demonstrate extensive nanoscale colocalization of JPH2 with RyR2 clusters in cardiomyocytes (18–20, 26). The functional significance of this arrangement was revealed by studies showing that JPH2 knockdown is associated with an increase in spontaneous Ca^2+^ spark frequency in cardiomyocytes (7) and that JPH2 overexpression reduces Ca^2+^ spark frequency (7, 27). These findings support the concept that direct interactions between JPH2 and RyR2 clusters reduce the open probability of RyR2s and decrease spontaneous Ca^2+^ spark frequency in cardiomyocytes (8). In the present study, we also observed nanoscale colocalization of JPH2 and RyR2 clusters near the plasma membrane of contractile SMCs. However, neither spontaneous Ca^2+^ spark frequency nor the amplitude and kinetics of individual Ca^2+^ spark events were altered by acute knockdown of JPH2 expression in these cells. The reason for this lack of a direct effect of JPH2 on RyR2 activity in SMCs is not immediately apparent, but may be related to differences in the structural arrangement of JPH2 and RyR2 clusters in cardiomyocytes compared with SMCs. A recent study using single-molecule DNA-PAINT and Exchange-PAINT superresolution modalities (28) demonstrated a high degree of coclustering of JPH2 and RyR2 in cardiomyocytes (26). These data suggest a model in which the excitability and Ca^2+^-signaling activity of RyR2 clusters in cardiac muscle is regulated by the intercalation of JPH2 protein clusters between individual RyR2 molecules within RyR2 protein clusters (26). The lack of an effect of JPH2 knockdown on Ca^2+^ spark frequency in SMCs reported here suggests that JPH2 and RyR2 do not form coclusters in these cells. However, the resolution of GSDIM is not sufficient to identify the position of individual RyR2 molecules within clusters relative to the position of JPH2 clusters; thus, further investigations applying Exchange-PAINT or other advanced imaging techniques to the study of SMCs are necessary to resolve this issue.

Our data showed that the primary functional consequence of JPH2 knockdown in SMCs is the loss of spontaneous, transient BK channel currents, suggesting that JPH2 is critically important for functional coupling of RyR2s and BK channels in contractile SMCs. These data indicate that the primary function of JPH2 in vascular SMCs is to maintain the proximity of the SR and plasma membrane, which serves to create microdomains that enable operation of the RyR2-BK channel Ca^2+^-signaling complex. The Ca^2+^ spark–BK channel pathway provides an important negative-feedback mechanism that limits the magnitude and duration of vasoconstrictor responses (12, 15, 29). Loss of this pathway following genetic knockout of the pore-forming alpha subunit or regulatory beta subunit of BK channels increases the sensitivity of the vasculature to vasoconstrictor stimuli, increasing total peripheral resistance and systemic blood pressure (30–32). Here, we found that knockdown of JPH2 expression had a similar effect, causing loss of spontaneous BK channel activity in SMCs and hypercontractility of cerebral and mesenteric resistance arteries, likely due to loss of functional coupling of RyR2 and BK channels. These data identify a critical role for JPH2 in vascular control.

Impaired JPH2 function and expression are associated with cardiac diseases (1, 2). For example, expression of JPH2 in the heart is decreased in animal models of dilated cardiomyopathy and hypertrophic cardiomyopathy associated with heart failure (33), and point mutations in *JPH2* are associated with hypertrophic cardiomyopathy (34–36) and arrhythmias (8) in human patients. The present study provides a conceptual framework for future investigations into the role of JPH2 in vascular diseases. Our findings predict that loss-of-function mutations or decreased expression of JPH2 in vascular SMCs would result in excessive arterial contractility and vascular resistance, potentially contributing to systemic hypertension, ischemic stroke, and vascular cognitive impairment and dementia.

## Materials and Methods

### Chemicals and Reagents.

All chemicals and reagents were obtained from Sigma-Aldrich unless stated otherwise.

### Animal Procedures and SMC Isolation.

All animal studies performed at the University of Nevada, Reno, were performed in accordance with institutional guidelines and received approval from the institutional animal care and use committee (IACUC), and those performed at University of Manchester were performed in accordance with the UK Home Office Guidance on the Operation of the Animals (Scientific Procedures) Act 1986, with the approval of an institutional review committee. Adult (12–16 wk) male C57Bl6 (Jackson Laboratory and Envigo) and smMHC^Cre/eGFP^ (Jackson Laboratory, stock no. 007742) transgenic reporter mice expressing enhanced green fluorescence protein (eGFP) under the control of the myosin heavy-chain promoter, which directs eGFP expression exclusively in SMCs (37), were used for this study. Mice were euthanized by decapitation and exsanguination under isoflurane anesthesia (at University of Nevada) or by CO_2_ followed by exsanguination (at University of Manchester). The brain and mesentery were isolated into a solution of ice-cold, Ca^2+^-free, Mg^2+^-based physiological saline solution (Mg-PSS) containing 5 mM KCl, 140 mM NaCl, 2 mM MgCl_2_, 10 mM Hepes, and 10 mM glucose (pH 7.4, NaOH). Cerebral pial and mesenteric resistance arteries were dissected from the brain and mesentery, respectively, and stored in this solution on ice. SMCs were isolated by digesting cerebral pial arteries in Mg-PSS supplemented with 1.0 mg/mL papain (Worthington Biochemical), 1 mg/mL dithioerythritol, and 10 mg/mL bovine serum albumin (BSA) at 37 °C for 12 min, washing 3 times with Mg-PSS, and then incubating a second time for 14 min at 37 °C in 1.0 mg/mL type II collagenase (Worthington Biochemical). SMCs were then liberated by triturating digested arteries. Isolated SMCs were stored in ice-cold Mg-PSS and were studied within 6 h. Whole brain, heart, and skeletal (quadriceps) muscle were also harvested from some mice for preparation of control samples for molecular studies.

### Isolation of SMCs Using Fluorescence-Activated Cell Sorting (FACS).

Native cerebral artery SMCs were isolated from smMHCCre/eGFP mice using a previously described FACS isolation protocol (17, 38). Briefly, cerebral arteries from smMHC^Cre/eGFP^ mice were enzymatically dispersed as described, and eGFP-expressing cells were sorted by FACS using a BD Biosciences FACSAria II flow cytometer (BD special order research product) with a 130-μm nozzle at a sheath pressure of 12 lb/in^2^. Cell viability was assessed using Hoechst 33258 (1 μg/mL) staining, with UV laser excitation at 355 nm and emission detection with a 450/50-nm bandpass filter. Cells with compromised membranes (positively stained with Hoechst 33258) were eliminated. Cells that were negative for Hoechst 33258 but positive for eGFP (488-nm excitation) were collected into a tube containing TRIzol reagent (Zymo) and further processed for RNA isolation. FACS was performed in the FACS/Flow Cytometry Shared Resource Laboratory (FCMSRL) Core Facility at the University of Nevada, Reno, School of Medicine.

### RNA Extraction and RT-PCR.

Total RNA, extracted using the TRIzol reagent, was purified from cerebral arteries, isolated cerebral artery SMCs, whole brain, and skeletal muscle using Direct-zol RNA Microprep (Zymo). Contaminating DNA was removed using OPTIZYME DNase I (Thermo Fisher Scientific), and first-strand cDNA was synthesized using qScript cDNA Supermix (Quanta Biosciences), as described by the manufacturers. Endpoint RT-PCR was performed using a T100 Thermal Cycler (Bio-Rad) with the indicated QuantiTect primers (Qiagen) spanning intron/exon boundaries of the following: *Jph1*, QT00134232 (Mm_Jph1_1_SG); *Jph2*, QT00126903 (Mm_Jph2_1_SG); *Jph3*, QT00164073 (Mm_Jph3_1_SG); and *Jph4*, QT00116725 (Mm_Jph4_1_SG). The custom primer pair 5′-CCA GCC TTC CTT CTT GGG TA-3′ (forward) and 5′-AGA GGT CTT TAC GGA TGT CAA CG-3′ (reverse) was used to amplify β-actin (*Actb*). All experiments included a template-free negative control. PCR products were resolved on 2% agarose gels containing ethidium bromide and imaged on a ChemiDoc system (Bio-Rad).

The relative abundance of *Jph1* and *Jph2* mRNAs in cerebral artery SMCs was determined by RT-qPCR, performed on a QuantStudio3 system (Thermo Fisher Scientific) using Fast SYBR Green master mix (Thermo Fisher Scientific) and the *Jph1*, *Jph2*, and *Actb* primer pairs listed here earlier. *Jph1* and *Jph2* C_t_ values were normalized to *Actb*, and mRNA expression levels were determined using the ΔΔCT method.

### Protein Extraction.

Cerebral arteries were dissected, cut into small segments, and placed directly into ice-cold 1× RIPA buffer (Cell Biolabs) supplemented with 1% protease mixture inhibitor (Cell Biolabs). Following a 15-min incubation, samples were disrupted using a hand-held homogenizer equipped with a sterile disposable tip (VWR) and incubated on ice for 15 min. Homogenized samples were centrifuged at 20,800 × *g* for 20 min at 4 °C, and the supernatant was transferred to a clean tube. The protein concentration of the supernatant was determined using a Pierce BCA Protein Assay Kit (Thermo Fisher), and absorbance values were read using a FlexStation 3 plate reader (Molecular Devices).

### Wes Protein Analysis.

JPH2 protein expression was measured using a Wes automated capillary‐based protein detection system (ProteinSimple) employing 25-capillary cartridges, 12 to 230-kDa Wes separation modules, and anti-rabbit detection modules, according to the manufacturer’s recommendations. Briefly, samples (0.1 to 0.5 mg/mL) were diluted in a fluorescence-reducing buffer and heated to 95 °C for 5 min before loading onto the Wes plate. A biotinylated ladder was included in all Wes experiments. Rows were successively loaded with Wes antibody diluent blocking buffer; anti-JPH2 (ab116077; Abcam) or anti–β-actin (ab8227, Abcam) primary antibodies, diluted 1:20 and 1:1,500, respectively; horseradish peroxidase (HRP)-conjugated anti-rabbit secondary antibody (1×; ProteinSimple); and a luminol–peroxide mix. Data were analyzed using Compass for SW (version 4.0; ProteinSimple).

### Knockdown of JPH2 Expression Using Morpholino Oligonucleotides.

The oligonucleotide used to specifically knock down JPH2 protein expression via steric inhibition of mRNA translation (morpholino) was designed and synthesized by Gene Tools. The sequence of the *Jph2*-targeting morpholino was 5′-TCA TCT CAT CCT CGC TCC TGA CAA C-3′. A nonsilencing morpholino (5′-CCT CTT ACC TCA GTT ACA ATT TAT A-3′; Gene Tools) with no known binding targets in rodents was used as a control for all experiments. After aseptically dissecting intact arteries from the brain or mesentery, morpholinos were delivered into SMCs by placing arteries in a 24-well plate containing 1 mL of serum‐free DMEM (Thermo Fisher Scientific) supplemented with Endo-Porter transfection reagent (6 µM; Gene Tools) and 10 µM control or *Jph2*-targeting morpholinos. Transfected cerebral arteries were cultured in this solution at 37 °C/5% CO_2_ for 48 h prior to experimentation. The efficiency of JPH2 knockdown was determined by comparing JPH2 protein expression levels in cerebral arteries treated with control and *Jph2*-targeting morpholinos using the Wes protein analysis system.

### Confocal Imaging of the SR and Plasma Membranes.

SMCs isolated from morpholino-treated arteries were allowed to adhere to 35-mm dishes (Corning; Fisher Scientific) for 1 h on ice. The SR and plasma membranes were labeled using a protocol that we previously described (10). SMCs were treated with ER-Tracker Green (5 μg/mL; Invitrogen) in Mg-PSS for 30 min at 37 °C, washed 3 times with Mg-PSS, and then treated with CellMask Deep Red (5 μg/mL; Invitrogen) for 5 min at 37 °C. Fluorescence images were obtained with an Andor Dragonfly 200 spinning-disk upright confocal microscope (Andor Technologies) using a 60× water-immersion objective (N.A. 0.9). Images were collected using a Zyla 4.2 Plus sCMOS camera. *Z*-stacks were acquired at 0.25-µm steps. Texas Red and fluorescein isothiocyanate were excited by illumination with 647- and 488-nm laser lines, respectively. All images were acquired at 2,048 pixels × 2,048 pixels and deconvolved using FUSION software (version 2.0; Oxford Instruments). Images were further processed and analyzed using Imaris (v.9.2) software (Bitplane). Lateral chromatic aberrations and astigmatism were determined using fluorescent microbeads (FocalCheck-TetraSpec; Fisher Scientific) and corrected during postprocessing. Deconvolved *Z*-stack images were imported and reconstructed, and each channel was thresholded. A third (colocalization) channel in which voxels were positive for both the red (plasma membrane) and green (SR) channel was created, and 3D surface plots were created for the plasma membrane, SR, and colocalization channels.

### GSDIM Superresolution Microscopy.

Marienfeld Superior no. 1.5 glass coverslips (no. 0107032) were sonicated in an aqueous solution of NaOH (5 M) for 1 h to remove fluorescent contaminants. Enzymatically isolated SMCs were seeded onto cleaned coverslips and fixed in 2% paraformaldehyde for 15 min at room temperature, washed with 1× phosphate-buffered saline (PBS; Gibco), permeabilized with 0.1% Triton X (Sigma-Aldrich), and blocked with 50% SEA Block (Thermo Fisher Scientific) in PBS. Cells were incubated overnight at 4 °C with primary antibodies against JPH2 (Thermo Fisher Scientific 40–5300, 1:400), RyR2 (Thermo Fisher Scientific MA3-916, 1:100), and/or BKα (Alomone Labs APC-021, 1:200) diluted in PBS supplemented with 20% SEA Block, 1% BSA, and 0.05% Triton X. Excess primary antibody was removed with a series of washes in 20% SEA Block solution. Alexa Fluor 568-conjugated goat anti-rabbit and/or Alexa Fluor 647-conjugated goat anti-mouse secondary antibodies (1:1,000; Life Technologies) were used for detection. Coverslips were mounted on glass depression slides using a thiol-based photo-switching imaging buffer consisting of 50 mM Tris/10 mM NaCl (pH 8), 10% glucose, 10 mM mercaptoethylamine, 0.48 mg/mL glucose oxidase, and 58 μg/mL catalase. To exclude oxygen and prevent rapid oxidation of the imaging buffer, coverslips were sealed to depression slides with Twinsil dental glue (Picodent).

Superresolution images were acquired in either epifluorescence or TIRF (150-nm penetration depth) illumination modalities using a GSDIM imaging system (Leica) equipped with an oil-immersion 160× HCX Plan-Apochromat (N.A. 1.47) objective, an electron-multiplying charge-coupled device camera (EMCCD; iXon3 897; Andor Technology), and 500-mW, 532- and 642-nm laser lines. Localization maps were constructed from images acquired at a rate of 100 Hz for 25,000 frames using Leica LAX software. Lateral chromatic aberrations and astigmatism corrections are integrated into the Leica GSDIM systems in parallel with the objective, the tube lens, and c-mount. Optimal image results are achieved through the interplay of these corrections. No other correction was applied to the resulting images. Postacquisition image analyses of cluster size distribution were performed using binary masks of the images in NIH ImageJ software. Object-based analysis was used to establish colocalization of JPH2 and RyR2 in superresolution localization maps. For this analysis, we used the JACoP colocalization analysis ImageJ plug-in, which applies a connexity analysis for image segmentation (23, 39).

The lateral spatial resolution of our GSDIM system was investigated using 40-nm nanorulers (GATTAquant) as described previously (40). Rulers consist of immobilized single-stranded DNA containing 3 binding sites for complimentary ATTO-655–labeled single-stranded DNA, each separated by 40 nm. Drift was corrected using fiducial reference markers incorporated in the nanorulers with the aid of GATTAquant analysis software. After plotting histograms of the distance separating dye molecules, we determined that the mean distance separating these molecules was 39.8 ± 12.3 nm. The full-width at half-maximum value of the point spread function was 20.7 ± 5.2 nm (*SI Appendix*, Fig. S4).

The specificity of anti-JPH2 and anti-RyR2 primary antibodies and Alexa Fluor-conjugated secondary antibodies used for GSDIM experiments was validated for superresolution microscopy in a prior study (26) and was further interrogated here by determining the labeling density (event counts per square micrometer) of superresolution images of SMCs. Cells incubated with anti-JPH2 primary antibody, its respective secondary antibody, and the secondary antibody corresponding to the anti-RyR2 primary antibody showed significantly higher event counts for JPH2 labeling than RyR2 labeling. When repeated replacing the anti-JPH2 primary antibody with an anti-RyR2 primary (*SI Appendix*, Fig. S5), the opposite was the case, demonstrating the specificity of our immunolabeling procedures.

### Ca^2+^ Imaging.

For Ca^2+^ spark recordings, cerebral arteries were transferred to Ca^2+^-free Mg-PSS containing the Ca^2+^ indicator dye Fluo-4-AM (10 µM) and pluronic acid (0.05%) and incubated in the dark for 45 min at room temperature. The arteries were then mounted on glass micropipettes in an arteriography chamber, pressurized to 60 mmHg, and superfused with imaging physiological saline solution (PSS) consisting of 119 mM NaCl_2_, 4.7 mM KCl, 21 mM NaCO_3_, 1.18 mM KH_2_PO_4_, 1.17 mM MgSO_4_, 0.026 mM EDTA, 1.8 mM CaCl_2_, and 4 mM glucose. Imaging PSS was aerated with a 5% CO_2_/21% O_2_/balance N_2_ gas mixture and warmed to 37 °C. Ca^2+^ sparks were imaged with an Andor spinning-disk confocal microscope system (Andor Technology). Following a 30-min equilibration period, Fluo‐4–loaded arteries were excited by illuminating at 488 nm using a solid‐state laser, and fluorescence emission was collected using a 527.5/49-nm bandpass filter. Images (131 × 131 μm) were acquired with Andor Revolution acquisition software every 18.9 ms (53 fps) using a 60× water-immersion objective (N.A. 1.0) and an electron-multiplying charge-coupled device camera (iXon 897; Andor Technologies) attached to a Nikon Eclipse TE‐2000U microscope. During postanalysis, Ca^2+^ sparks, defined as temporally delineated increases in fractional fluorescence (*F*) greater than 1.26-fold above baseline fluorescence levels (*F*_0_) in defined regions of interest (ROIs) of 1.5 × 1.5 μm were automatically detected using SparkAn software. The frequency of Ca^2+^ sparks (in Hertz), as well as the amplitude (*F*/*F*_0_), duration, and rise and decay times of each individual event, were determined from these recordings.

The same preparation was used to record the effects of *Jph2* knockdown on global Ca^2+^ signals stimulated by caffeine or UTP. For these experiments, arteries were preincubated with wortmannin (1 µM, 45 min) to reduce contractility. Caffeine (10 mM) or UTP (30 µM) was added as a bolus directly to the arteriograph chamber while maintaining constant perfusion with PSS. Caffeine or UTP pulses were repeated after a 4-min incubation period. Changes in *F*/*F*_0_, time to peak response, and response duration for an ROI of 70 × 160 pixels (10 to 15 cells) were determined using SparkAn software.

### Patch-Clamp Electrophysiology.

All currents were recorded using an AxoPatch 200B amplifier equipped with an Axon CV 203BU headstage (Molecular Devices). Currents were filtered at 1 kHz, digitized at 40 kHz, and stored for subsequent analysis. Clampex and Clampfit (version 10.2; Molecular Devices) were used for data acquisition and analysis, respectively. All recordings were performed at room temperature (22 °C). SMCs isolated from morpholino-treated arteries were transferred to a recording chamber and allowed to adhere to glass coverslips for 10 min at room temperature. Recording electrodes (3 to 5 MΩ) were pulled and polished. For perforated-patch whole-cell recordings, amphotericin B (40 µM) was included in the pipette solution to allow electrical access. Perforation was deemed acceptable if series resistance was less than 40 MΩ. STOCs were recorded in a bathing solution containing 134 mM NaCl, 6 mM KCl, 1 mM MgCl_2_, 2 mM CaCl_2_, 10 mM Hepes, and 10 mM glucose at pH 7.4 (NaOH). The pipette solution contained 110 mM K-aspartate, 1 mM MgCl_2_, 30 mM KCl, 10 mM NaCl, 10 mM Hepes, and 5 µM EGTA at pH 7.2 (NaOH). STOCs were recorded from SMCs voltage-clamped at a range of membrane potentials (−60 to 0 mV).

Whole-cell K^+^ currents were recorded using a step protocol (−100 to +100 mV in 20-mV steps for 500 ms) from a holding potential of −80 mV. Whole-cell BK currents were isolated by current subtraction following administration of the selective BK channel blocker paxilline (1 µM). Current–voltage (I–V) plots were generated using currents averaged over the last 50 ms of each voltage step. The bathing solution contained 134 mM NaCl, 6 mM KCl, 10 mM Hepes, 10 mM glucose, 2 mM CaCl_2_, and 1 mM MgCl_2_ at pH 7.4 (NaOH). The pipette solution contained 140 mM KCl, 1.9 mM MgCl_2_, 75 µM Ca^2+^, 10 mM Hepes, 0.1 mM EGTA, and 2 mM Na_2_ATP at pH 7.2 (KOH).

### Pressure Myography.

Cerebral and mesenteric arteries treated with *Jph2*-targeting and control morpholinos were studied ex vivo using pressure myography. Arteries were mounted between 2 glass pipettes (outer diameter, ∼40 to 50 μm) in a pressure myograph chamber (Living Systems Instrumentation) and secured with nylon monofilaments. Intraluminal pressure was controlled using a servo-controlled peristaltic pump (Living Systems Instrumentation). Pressurized arteries were visualized with an inverted microscope (Accu-Scope) coupled to a USB camera (The Imaging Source). Intraluminal diameter as a function of time was acquired using IonWizard software (version 6.4.1.73; IonOptix). Arteries were bathed in warmed (37 °C), oxygenated (21% O_2_/6% CO_2_/73% N_2_) PSS (119 mM NaCl, 4.7 mM KCl, 21 mM NaHCO_3_, 1.17 mM MgSO_4_, 1.8 mM CaCl_2_, 1.18 mM KH_2_PO4, 5 mM glucose, 0.03 mM EDTA) at an intraluminal pressure of 5 mmHg. After a 15-min equilibration period, intraluminal pressure was increased to 110 mmHg and vessels were stretched to their in vivo length, after which pressure was reduced back to 5 mmHg for an additional 15 min. The viability of each preparation was verified by measuring constriction in response to bath application of PSS containing 60 mM K^+^ (high-extracellular [K^+^] PSS), made isotonic by adjusting the NaCl concentration (63.7 mM). Arteries that showed less than 10% constriction in response to elevated K^+^ were deemed to have been damaged during isolation or cannulation and were excluded from analysis.

Myogenic reactivity was assessed by lowering the intraluminal pressure to 5 mmHg for 15 min and then slowly raising it in stepwise fashion to 140 mmHg in 20-mmHg increments. This procedure was performed twice, first in Ca^2+^-containing PSS with the composition indicated here earlier (active diameter), and subsequently in arteries superfused with Ca^2+^-free PSS supplemented with EGTA (2 mM) and the voltage-dependent Ca^2+^ channel blocker diltiazem (10 μM) to inhibit SMC contraction (passive diameter). Lumen diameter was continuously recorded, and vessels were allowed to equilibrate at each pressure step until a steady-state diameter was reached. Myogenic tone at each pressure step was calculated as myogenic tone (percentage) = [1 − (active lumen diameter/passive lumen diameter)] × 100. In separate experiments, control vessels were treated with the RyR blocker tetracaine (10 µM), added to the bathing solution, before assessing myogenic reactivity.

## Supplementary Material

Supplementary File

Supplementary File

Supplementary File

Supplementary File

Supplementary File
