# Supplementary material for: Nanoscale coupling of junctophilin-2 and ryanodine receptors regulates vascular smooth muscle cell contractility
Source: Proc Natl Acad Sci U S A. 2019 Oct 7;116(43):21874–81. doi: 10.1073/pnas.1911304116 (PMC6815135; doi:10.1073/pnas.1911304116)
Supplement: Supplementary File [file pnas.1911304116.sapp.pdf]

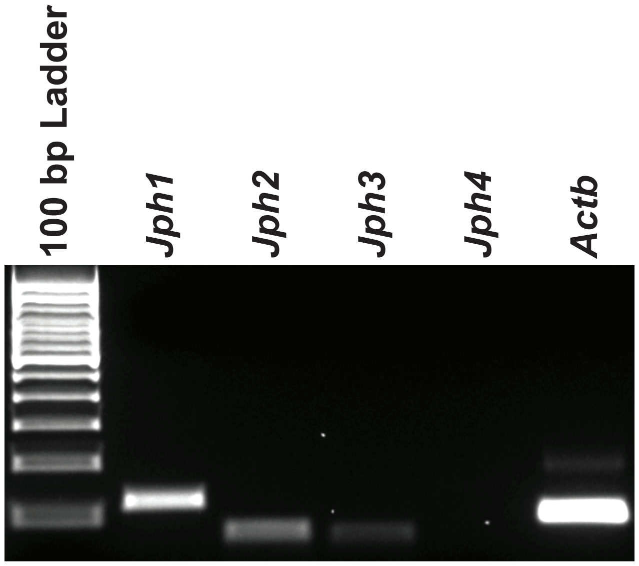

Supplemental figure S1. *Jph* isotype expression in mesenteric arteries. Representative end-point RT-PCR analysis for expression of *Jph1*, *Jph2*, *Jph3*, and *Jph4* in RNA samples isolated from whole mesenteric arteries (n = 3 independent experiments). β-actin (*Actb*) was used as a positive control.

www.pnas.org/cgi/doi/10.1073/pnas.1911304116

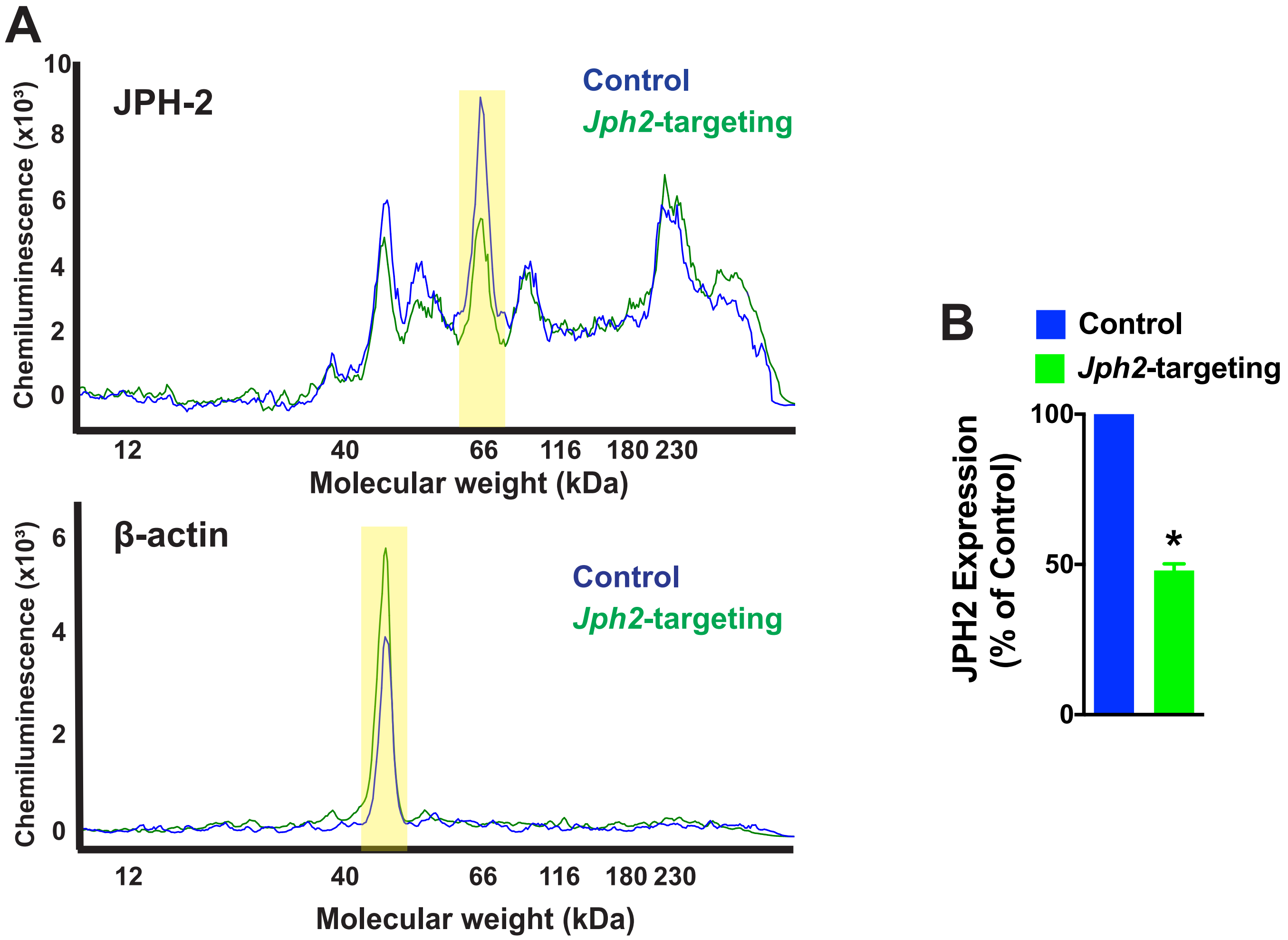

Supplemental figure S2. Knockdown of JPH2 in cerebral arteries. A) Representative electropherogram from a Wes capillary electrophoresis protein analysis experiment showing chemiluminescence intensity peaks as a function of molecular weight (kDa) for protein lysates isolated from cerebral arteries treated with control (blue) or *Jph2*-targeting (green) morpholinos and probed with primary antibodies against JPH2 (top) or β-actin (bottom). B) Summary data showing JPH2 chemiluminescence normalized to β-actin chemiluminescence for protein lysates isolated from cerebral arteries treated with control (blue) or *Jph2*-targeting (green) morpholinos. (N = 3 animals/group; *P < 0.05).

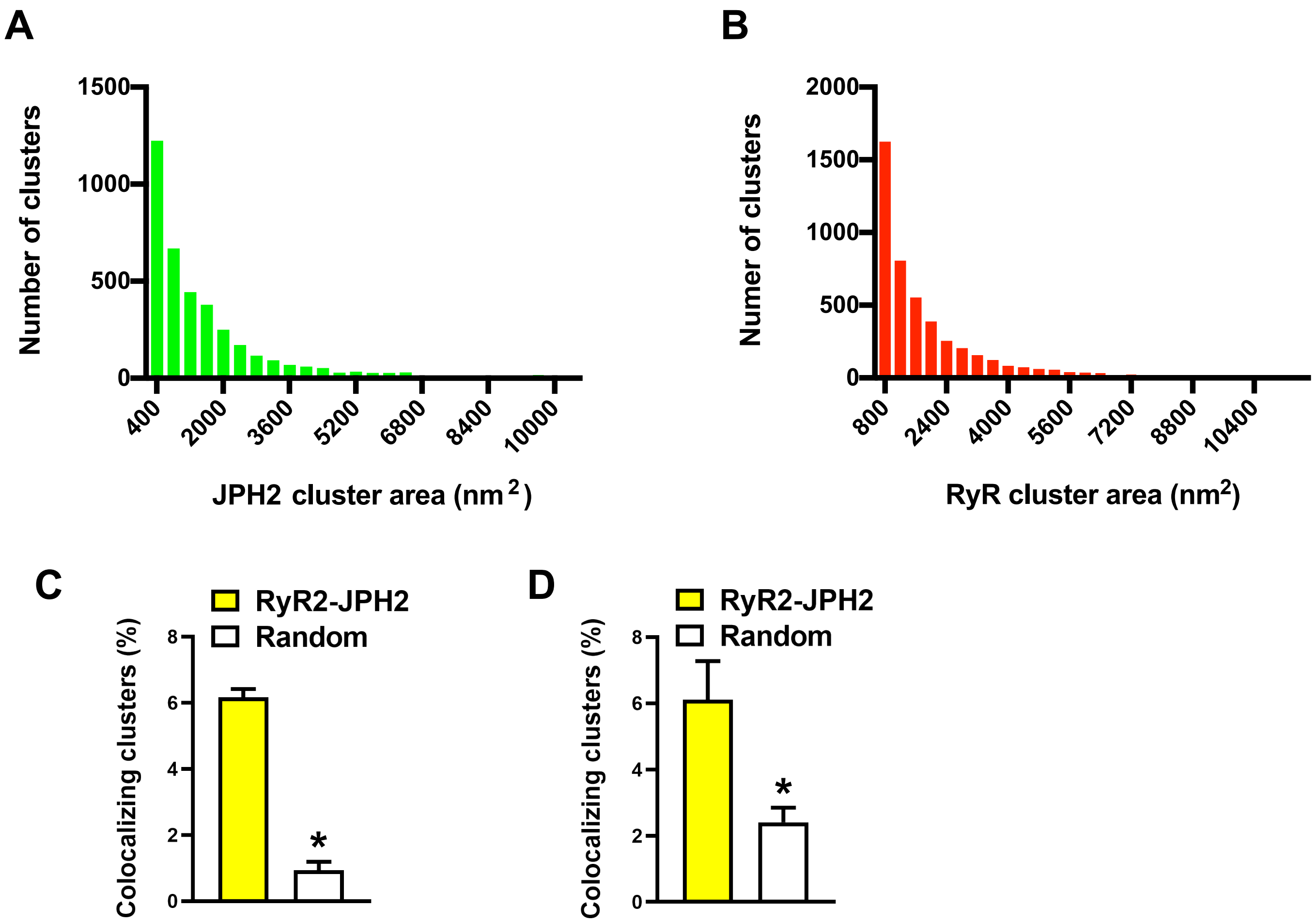

Supplemental figure S3. JPH2 and RyR2 cluster size distribution and colocalization. A) Histogram showing exponential size distribution of JPH2 protein clusters (n = 9 cells, 3888 clusters). B) Histogram showing exponential size distribution of RyR2 protein clusters (n = 9 cells, 4686 clusters). C and D) Object-based analysis comparing the fraction of JPH2 and RyR2 colocalizing clusters with the fraction of clusters that colocalize to a random distribution of clusters, imaged in epifluorescence (EPI) (C) or TIRF (D) illumination modalities. JPH2-RyR2: 6.17% ± 0.24% (EPI) and 6.12% ± 1.16% (TIRF); Random: 0.94% ± 0.25% (EPI) and 2.41% ± 0.44% (TIRF). *P < 0.05. Data are expressed as means ± SEM (n = 7–8 cells from 3 animals).

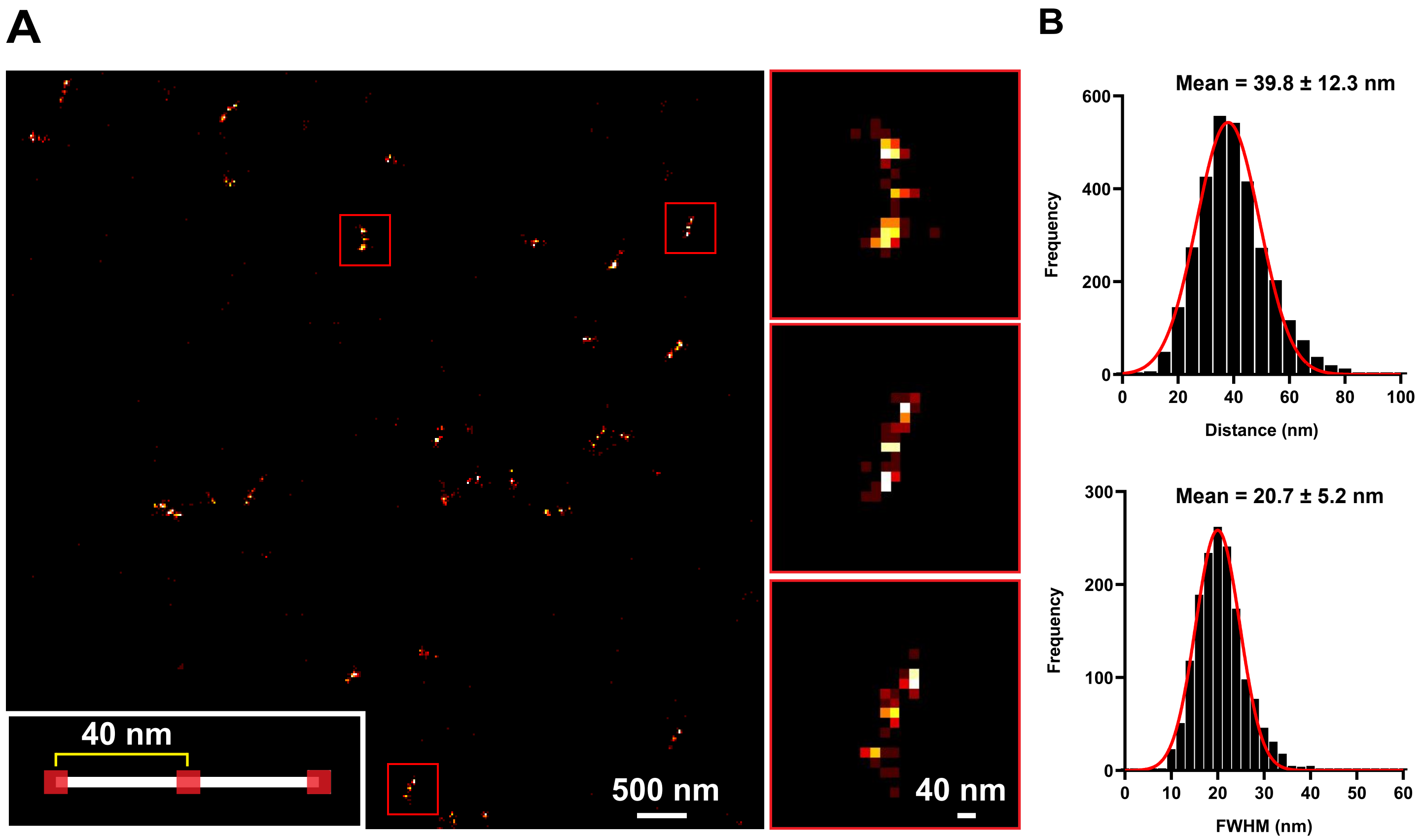

Supplemental figure S4. Localization accuracy of GSDIM. A) Super-resolution localization map of GATTA-PAINT 40RG nanorulers in the red channel (ATTO 655). The inset (bottom left) shows a diagram of the GATTA-PAINT 40RG nanoruler, containing three ATTO 655 and ATTO 542 fluorophore binding sites spaced 40 nm apart. The three ROI images on the right show zoomed-in sections of individual nanorulers. B) Frequency distribution data of the distance between fluorophores (n = 3158) and full-width half-maximum (FWHM) value for each individual fluorophore (n = 1579). Data are expressed as means ± SD.

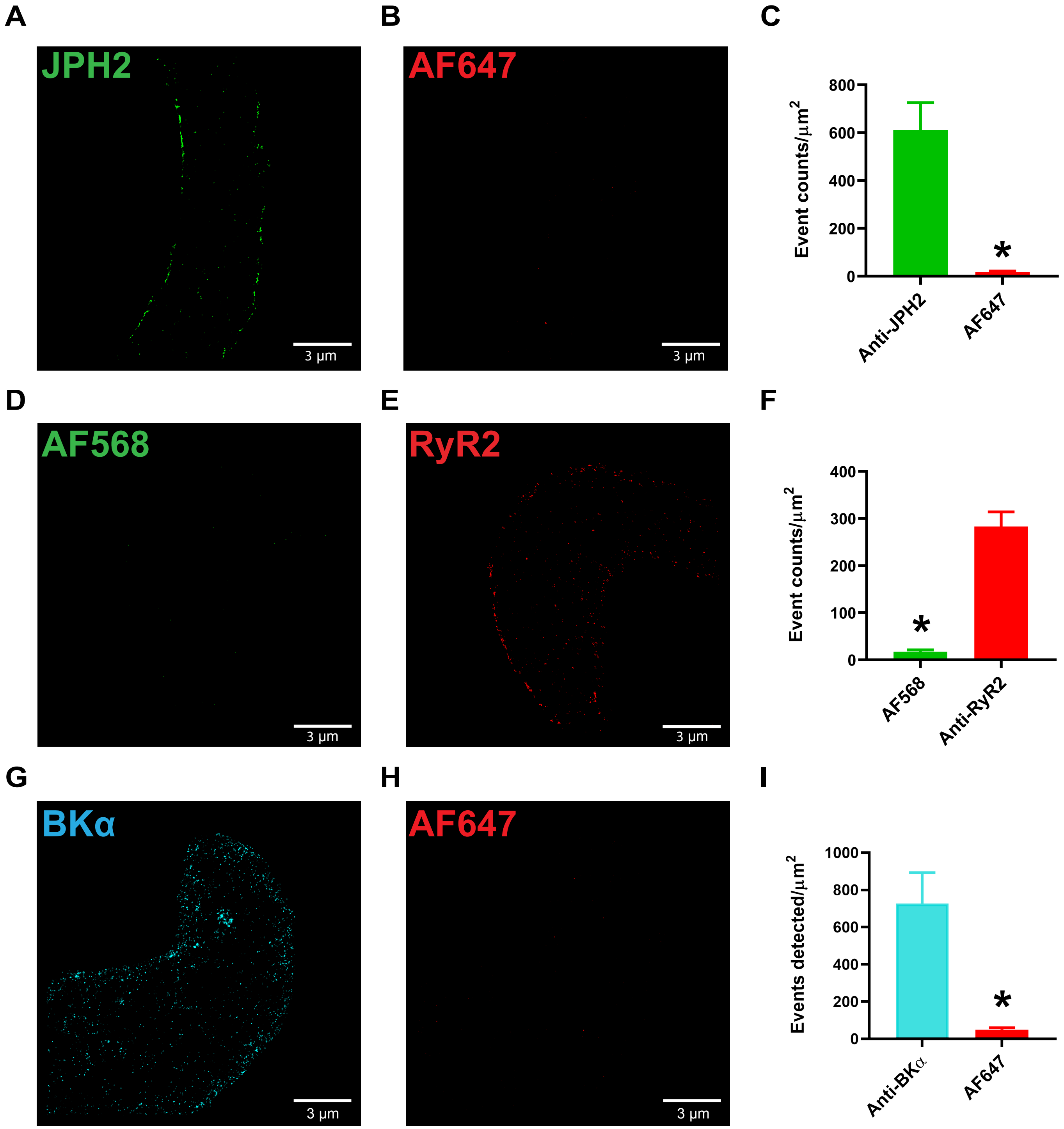

Supplemental figure S5. JPH2, RyR2, and BKα antibody specificity. A) Super-resolution localization map for JPH2 (green) and B) Alexa Fluor 647 secondary-only (red) in native cerebral artery SMCs imaged with GSDIM using the epifluorescence illumination mode. C) Summary data showing event counts corresponding to localization maps in panels A and B (n = 8 cells from 3 animals; *P < 0.05). Data are expressed as means ± SEM. D and E) Super-resolution localization map similar to A and B, but for RyR2 (red) and Alexa Fluor 568 secondary (green) only. Scale bar = 3 µm. F) Summary data showing event counts corresponding to localization maps in panels D and E (n = 8 cells from 3 animals; *P < 0.05). G) Super-resolution localization map for BKα (Cyan) and H) Alexa Fluor 647 secondary only (red). Scale bar = 3 µm. I) Summary data showing event counts corresponding to localization maps in panels G and H (n = 8 cells from 3 animals; *P < 0.05). Data are expressed as means ± SEM.

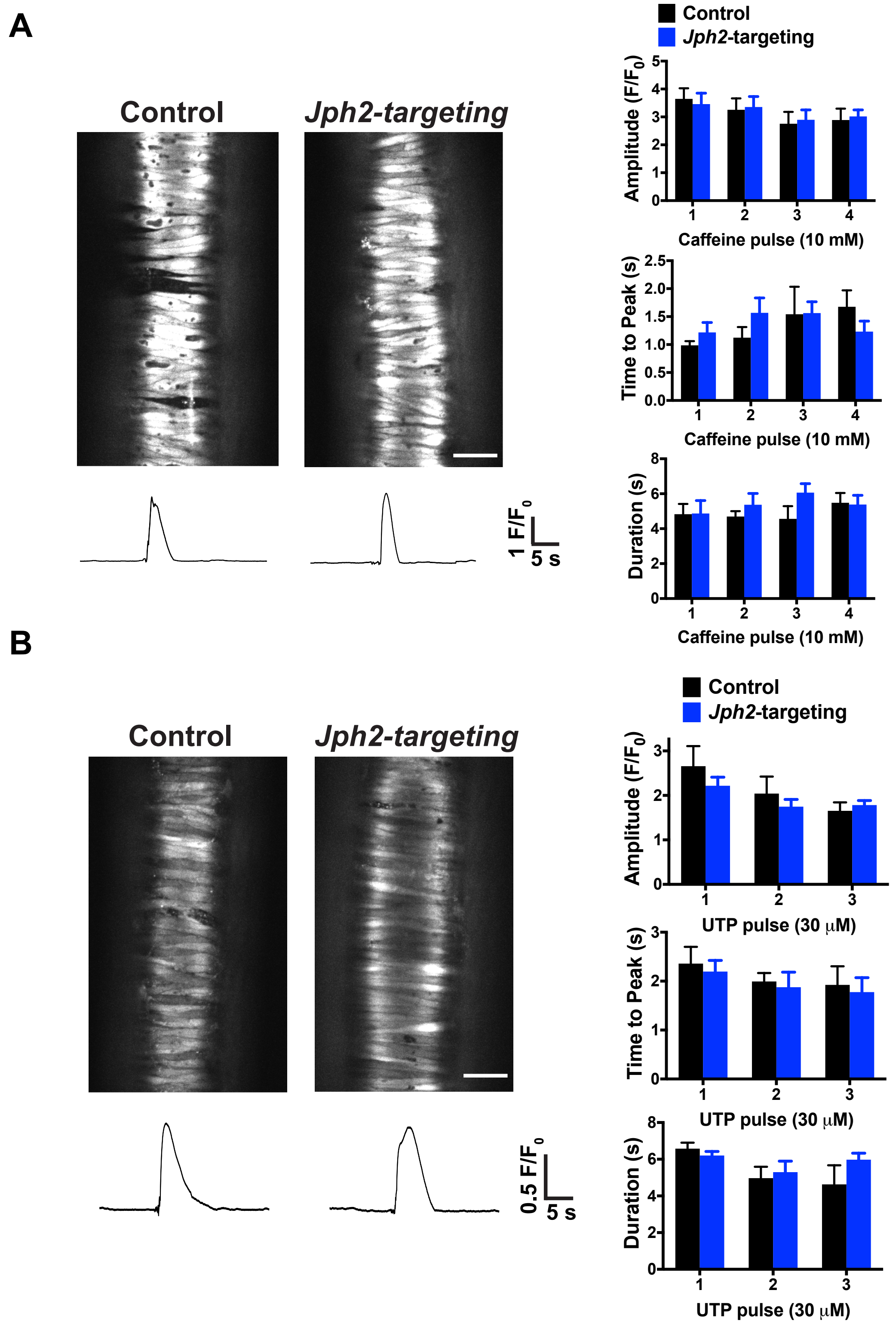

Supplemental figure S6. Jph2 knockdown does not alter total SR Ca^{2+} store load, IP_3R-mediated Ca^{2+} release, or SR Ca^{2+} uptake. A) Images on the left show pressurized cerebral arteries treated with control or *Jph2*-targeting morpholinos and exposed to a bolus dose of caffeine (10 mM). Scale bar = 50 µm. Below: Representative traces showing the increase in fractional fluorescence (F/F_0) as a function of time following application of caffeine for a region of interest (ROI) in the corresponding image encompassing 10–15 cells. Right: Summary data showing the amplitude, time to peak response, and duration of caffeine-induced Ca^{2+} transients in arteries treated with control or *Jph2*-targeting morpholinos over four separate applications of caffeine (n = 6–7 arteries, from 3–4 mice/group). No significant differences were detected. B) Images on the left show pressurized cerebral arteries treated with control or *Jph2*-targeting morpholinos and exposed to a bolus dose of UTP (30 µM). Scale bar = 50 µm. Below: Representative traces showing the increase in fractional fluorescence (F/F_0) as a function of time following application of caffeine for an ROI in the corresponding image encompassing 10-15 cells. Right: Summary data showing the amplitude, time to peak response, and duration of UTP-induced Ca^{2+} transient in arteries treated with control or *Jph2*-targeting morpholinos over three separate applications of UTP (n = 5–6 arteries from 5 mice/group). No significant differences were detected.

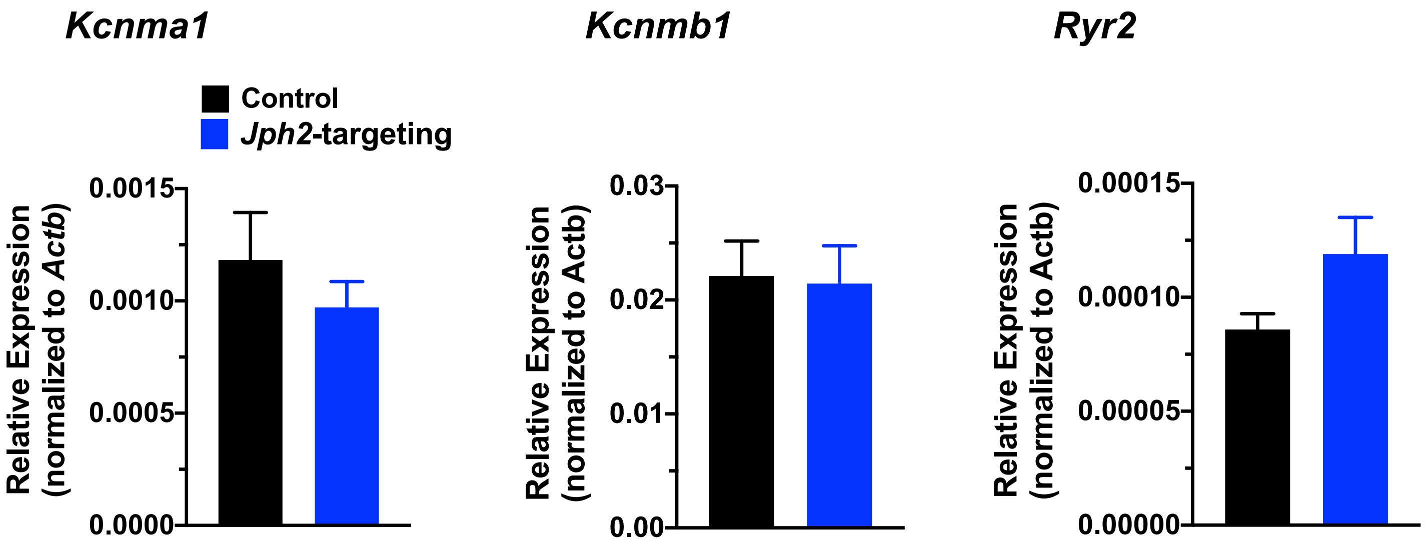

Supplemental figure S7. ***Jph2*** **knockdown does not alter expression levels of RyR2, BKα, or BKβ1**. Quantitative RT-PCR analysis of the expression of the BKα-subunit encoded by *Kcnma1*, BKβ1-subunit encoded by *Kcnmb1*, and *Ryr2* in RNA samples isolated from whole cerebral arteries (CA) treated with control or *Jph2*-targeting morpholinos. Expression levels are normalized to those of the housekeeping gene β-actin (Actb) (n = 3 animals per group).

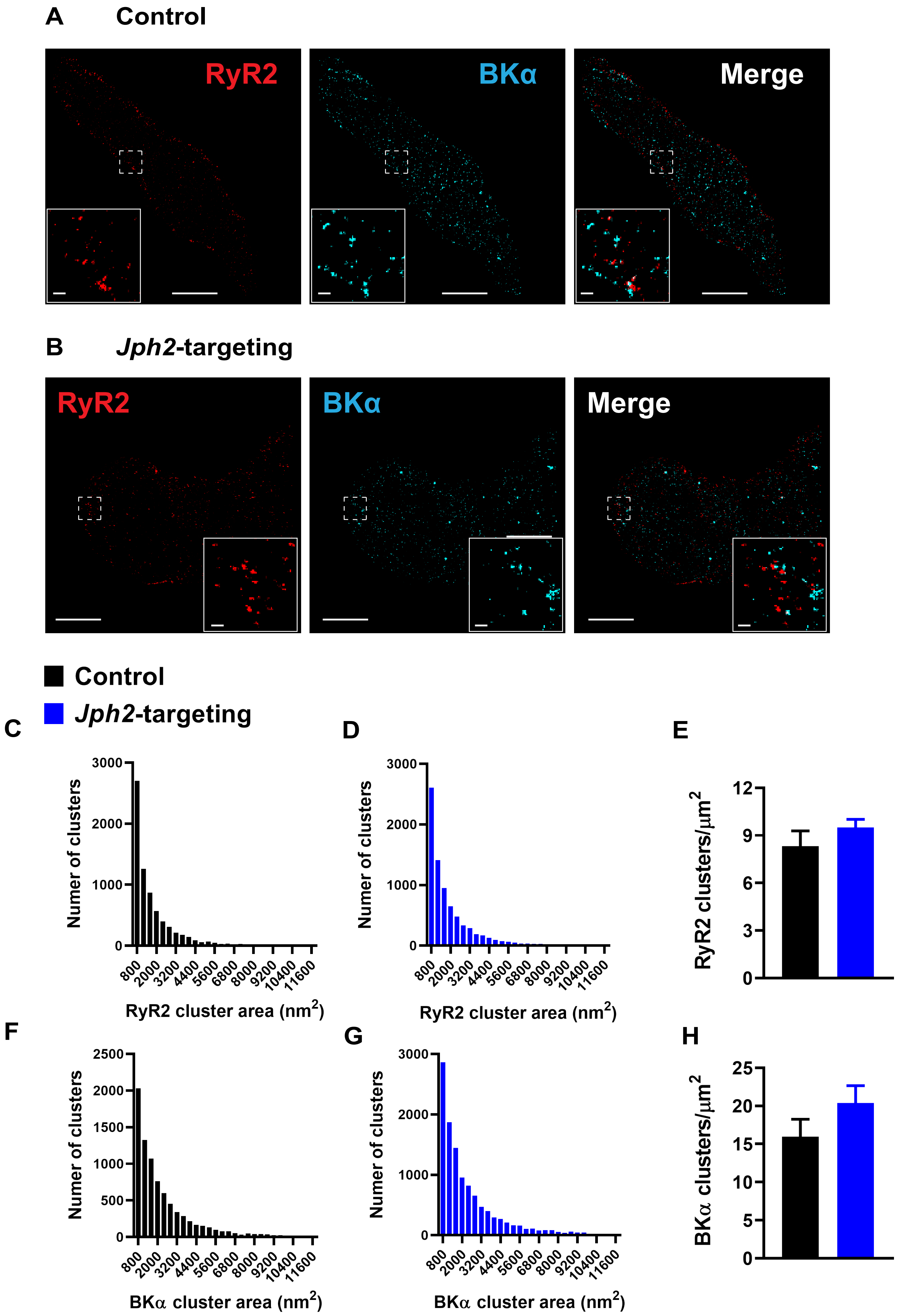
A
Control
RyR2
BKα
Merge
B
Jph2-targeting
RyR2
BKα
Merge
Control
Jph2-targeting
C
Numer of clusters
RyR2 cluster area (nm2)
D
Numer of clusters
RyR2 cluster area (nm2)
E
RyR2 clusters/μm2
F
Numer of clusters
BKα cluster area (nm2)
G
Numer of clusters
BKα cluster area (nm2)
H
BKα clusters/μm2

Supplemental figure S8. *Jph2* knockdown does not alter RyR2 or BKα protein cluster size distribution or density. Super-resolution localization maps for native cerebral artery SMCs immunolabeled for RyR2 (red) and BKα (cyan), treated with control (A) or *Jph2*-targeting morpholinos (B), and imaged using GSDIM in epifluorescence illumination mode. Merged images are also shown. Scale bars = 3 µm. Insets: Expanded view of the white rectangle in each image. Scale bars = 0.2 µm. Maps are representative of n = 8 cells from 3 animals. Histograms showing the size distribution of RyR2 clusters in cells treated with (C) control (7228 clusters) or (D) *Jph2*-targeting (7836 clusters) morpholinos (n = 8 cells), and (E) the density of RyR2 clusters in both groups (n = 8 cells). Histograms showing the size distribution of BKα clusters in cells treated with (F) control (13,918 clusters) or (G) *Jph2*-targeting (18,156 clusters) morpholinos (n = 8 cells), and the density of BKα clusters in both groups (n = 8 cells).

SI Movie Legends

Movie 1. SR and plasma membrane interactions in a SMC isolated from a cerebral artery treated with control morpholinos. Animated representation of a deconvolved z-stack confocal image series that was reconstructed and rendered in 3D. The plasma membrane is shown in red, the SR is shown in green, and areas of colocalization are shown in yellow.

Movie 2. SR and plasma membrane interactions in a SMC isolated from a cerebral artery treated with *Jph2*-targeting morpholinos. Animated representation of a deconvolved z-stack confocal image series that was reconstructed and rendered in 3D. The plasma membrane is shown in red, the SR is shown in green, and areas of colocalization are shown in yellow.

Movie 3. Ca^{2+} sparks in a cerebral artery treated with control morpholinos. Representative time series spinning-disk confocal images of dynamic Ca^{2+} signals in a pressurized (60 mmHg) cerebral artery treated with control morpholinos.

Movie 4. Ca^{2+} sparks in a cerebral artery treated with *Jph2*-targeting morpholinos. Representative time series spinning-disk confocal images of dynamic Ca^{2+} signals in a pressurized (60 mmHg) cerebral artery treated with *Jph2*-targeting morpholinos.
